# STAT6 Mediates Footpad Immunopathology in the Absence of IL-12p40 Following Infection of Susceptible BALB/c Mice With *Leishmania major*

**DOI:** 10.3389/fimmu.2018.00503

**Published:** 2018-03-14

**Authors:** Florence Kauffmann, Elyn Meert, Kaat de Jonge, Yvon Elkrim, Delphine Hanot Mambres, Olivier Denis, Eric Muraille, Stefan Magez, Carl De Trez

**Affiliations:** ^1^Laboratory of Cellular and Molecular Immunology, Vrije Universiteit Brussel (VUB), Brussels, Belgium; ^2^Laboratory of Myeloid Cell Immunology, VIB-UGent Center for Inflammation Research (IRC), Ghent, Belgium; ^3^Unité de Recherche en Biologie des Microorganismes, Laboratoire d’Immunologie et de Microbiologie, Université de Namur, Namur, Belgium; ^4^Scientific Service Immunology, Scientific Institute of Public Health (WIV-ISP), Brussels, Belgium; ^5^Laboratoire de Parasitologie, Université Libre de Bruxelles (ULB), Brussels, Belgium; ^6^Ghent University Global Campus, Incheon, South Korea

**Keywords:** BALB/c mice, IL-12p40^−/−^, STAT6^−/−^, *Leishmania major*, immunopathology, neutrophils

## Abstract

*Leishmania major* (*L. major*) parasites are intracellular parasites belong to the *Trypanosomatidae* family and are the causative agent of cutaneous leishmaniasis. This disease affects approximately 1.5 million per year worldwide and there is currently no prophylactic vaccine available. *L. major* is transmitted by the bite of an infected sandfly and has been considered for decades now as a mouse model of choice to identify the factors implicated in T helper (Th)1 and Th2 polarization due to the natural resistance and susceptibility to infection of C57BL/6 and BALB/c mice, respectively. In this study, we refine the role of IL-12p40 cytokine, which is implicated the development of a protective Th1 response, and STAT6, a transcription factor involved in the signaling *via* detrimental interleukin (IL)-4 and IL-13 associated Th2 cytokines during *L. major* infection in the BALB/c model. In the absence of STAT6 and IL-12p40 signaling, double knockout (DKO) susceptible BALB/c mice displayed reduced footpad swelling and ulcerative lesion compared to IL-12p40^−/−^ mice upon *L. major* infection. Hence, they expressed slower upregulation of keratinocyte markers implicated in the inhibition of wound healing, such as keratin 6a (Krt6a) and Krt16. This coincides with the presence of neutrophils displaying an altered phenotype characterized by a lower expression of surface markers Ly6C, CD11b, and Ly6G. These neutrophils exhibited very lower levels of apoptosis similarly to neutrophils present in resistant STAT6^−/−^ mice. Interestingly, the reduced footpad swelling in DKO mice is associated with a high footpad parasite level similar to susceptible IL-12p40^−/−^ mice. In conclusion, this study demonstrate that in the absence of both STAT6 and IL-12p40 signaling, *L. major*-infected mice display smaller and less ulcerated lesions, which does, however, not correlate with reduced parasite load. In addition, the presence of neutrophils with an altered phenotype is associated with reduced apoptosis and delayed immunopathologies, demonstrating the detrimental role of STAT6 in infected susceptible BALB/c mice.

## Introduction

Leishmaniasis is a neglected tropical disease caused by the bite of *Leishmania*-infected phlebotomine sandflies which affects both humans and animals. Several forms of the human disease exist, going from localized and self-healing cutaneous lesions in the case of cutaneous leishmaniasis (CL) to visceralizing and potentially fatal form of the disease in case of visceral leishmaniasis. An estimated 12 million people are affected by *Leishmania* parasites with ±1.5 million and ±0.5 million new cases of CL and CL occuring per year, respectively. Clinical manifestations of the disease vary upon the parasite species transmitted and the type of infected host, its genetic background and health status. *Leishmania major* causes the cutaneous form of the disease, mostly infects humans in the Middle East and has been used extensively in murine models. The genetic predisposition toward resistance to *L. major* murine infection is classically associated with an interleukin-12 (IL-12) driven, interferon-γ (IFN-γ)-dominated T helper (Th)1 response, which leads to the active killing of parasites inside macrophages by the action of nitric oxide, tumor necrosis factor-α, and reactive oxygen species. Conversely, susceptibility to murine *L. major* infections is related to an IL-4/IL-13-induced Th2 response which causes disease and can be fatal (1). Recently, however, this Th1/Th2 dichotomy model has been challenged by the discovery of other immune players, such as Th17, Treg and Th19 cells, IL-10 and B cells, playing a role in resistance/susceptibility to *L. major* infection (2).

Neutrophils are innate immune cells that are rapidly recruited from the bone marrow *via* the blood to the site of infection. They express a Ly6G^+^ CD11b^+^ Ly6C^int^ phenotype and play an essential role in the elimination of various pathogens, such as bacteria, viruses, fungi, and parasites (3–6). Besides being phagocytizing, neutrophils have been shown to regulate the innate and adaptive components of the immune system (7). During *L. major* infections, neutrophils are the first cells to massively arrive at the site of *L. major* infection, internalize parasites, which are subsequently taken up by a second wave of recruited monocytes (8, 9). Additionally, neutrophils display immunomodulatory functions during *L. major* infections, being disease promoting or host protective depending on the host genetic background (10–13). Also, neutrophils can play a detrimental role in the development of immunopathology in the absence of IL-10 during CL (14).

IL-12p40 is a cytokine subunit which, in composition with IL-12p35, forms the functional heterodimer IL-12p70. IL-12p40 can also dimerize with an IL-12p35-related subunit, IL-12p19, to form IL-23 (15). During *L. major* infections, both IL-12p35 and IL-12p40 have been shown to be essential for inducing a Th1 response in mice *via* Signal Transducer and Activator of Transcription (STAT)4 signaling (16–18). Homodimers of the IL-12p40 subunit, resulting IL-12p80, has been associated with susceptibility to *L. major* infections in BALB/c mice. Signal Transducer and Activator of Transcription (STAT) 6 is a protein which in response to IL-4 and IL-13 is required for the development of a Th2 response (19). The inhibitory role of STAT6 on inducing resistance during CL has been demonstrated using STAT6^−/−^ mice. As such, *Leishmania mexicana*-infected STAT6^−/−^ mice show resistance by mounting a Th1 response and *L. major*-infected STAT6^−/−^ mice have higher levels of anti-parasitic iNOS-producing dendritic cells (20, 21). The present study addresses the fundamental question of what the impact would be on the pathological outcome of *L. major* IR75 infection in the absence of both the IL-12p40 and the STAT6 signaling.

Together, our results demonstrate that in the absence of both IL-12p40 and STAT6 signaling, infected BALB/c mice display reduced footpad swelling and ulceration, but similar parasite loads as compared to IL-12p40-deficient mice. This phenotype is associated with delayed increase of specific keratinocyte markers implicated in the inhibition of wound healing, e.g., keratin 6a (Krt6a) and 16, compared to IL-12p40-deficient mice and to the recruitment of neutrophils expressing lower levels of Ly6C, CD11b, and Ly6G and displaying less apoptosis. To the whole, these results pointed out the detrimental role of STAT6 in *L. major*-induced immunopathologies in the absence of IL-12p40.

## Materials and Methods

### Ethic Statements

All the experiments were performed according to directive 2010/63/EU of the European Parliament for the protection of animals used for scientific purposes and approved by the Ethical Committee for Animal Experiments of the Vrije Universiteit Brussel (clearance number 14-220-18).

### Animals and Parasites

Wild-type BALB/c mice were acquired from Harlan (Bicester, UK). STAT6^−/−^ BALB/c mice (strain C.129S2-STAT6tm1Gru/J) and IL-12p40^−/−^ BALB/c mice (C.129S1-IL12btm1Jm/J) were purchased from the Jackson Laboratory. The double knockout (DKO) STAT6^−/−^IL-12p40^−/−^ BALB/c mice were obtained by a cross between the two single-gene knockout strains cited above (1). All mice were housed at the animal facility at the Vrije Universiteit Brussel in filter-top cages containing a maximum of seven mice, with enriched environment and unrestricted access to food and water. *L. major* IR75 (MRHO/IR/75/ER) parasites were grown in M199 medium (GIBCO) supplemented with 20% fetal calf serum (FCS), 100 U/ml penicillin (GIBCO), 100 µg/ml streptomycin (GIBCO), 4 mM NaHCO_3_ (Merck), 0.0005% hemin (Sigma-Aldrich), and 0.1 mM adenosine (Sigma-Aldrich).

### Infections and Treatment

*Leishmania major* parasites were harvested from the footpad-draining lymph nodes of *L. major*-infected BALB/c mice (after 3–4 weeks of infection). After 6 days of culture growth in M199 medium supplemented with 20% FCS, 100 U/ml penicillin (GIBCO), 100 µg/ml streptomycin (GIBCO), 4 mM NaHCO_3_ (Merck), 0.0005% hemin (Sigma-Aldrich), and 0.1 mM adenosine (Sigma-Aldrich), the parasites were harvested by centrifugation (3,000 rpm, 10 min, 20°C) and washed in DMEM (GIBCO) medium. The metacyclic promastigotes were purified using a polysucrose (Sigma-Aldrich, St. Louis, MO, USA) gradient (20 and 10% in DMEM) by centrifugation (2,500 rpm, 15 min, 20°C, no break). Thereafter, the purified metacyclic promastigotes were washed twice in DMEM medium before being used for infection. The mice were infected with 2 × 10^6^ *L. major* IR75 parasites in the hind footpads. The swelling of footpads of naïve and infected mice was monitored two times weekly using a Vernier caliper (Mitutoyo). Simultaneously, photographs of the naïve and infected footpads (front and side) were taken. The mice were sacrificed at indicated times by the use of CO_2_ gas. From day 0, DKO mice were treated daily for a week with 1 µg of recombinant murine IL-12p70 (PeproTech EC, Ltd., London, UK) *via* the intraperitoneal route.

### Footpad Homogenization

Footpad lesions of infected mice were cut tangentially to the bone and mechanically disrupted with surgical scissors in cold RPMI (GIBCO) medium containing 100 U/ml Collagenase III (Worthington Biochemical Corporation, Lakewood, NJ, USA) and 50 U/ml DNase I (Worthington Biochemical Corporation, Lakewood, NJ, USA). The disrupted footpads were then incubated in this enzyme mixture for 30 min at 37°C. The reaction was stopped using cold RPMI medium with 2.5 mM EDTA (Duchefa Biochimie) and filtered over a 70 µm nylon filter mesh. The single cell suspension was centrifuged (1,400 rpm, 7 min, 4°C) and resuspended in PBS + 5% FCS.

### Parasitemia Determination

The viable parasite burden in the footpads was determined by limiting dilutions as described elsewhere (22). Single cell suspensions were prepared as described above and serial dilutions (twofold) were pipetted across a flat-bottom 96-well plate with three replicates for each footpad in an end-point titration. The plates were incubated for 10–14 days at 26°C before the number of *Leishmania*-positive wells was determined under an inverted light microscope.

### Flow Cytometry

Single cell suspensions of footpad cells were filtered over a 70-µm nylon filter mesh and incubated in saturating doses of purified anti-mouse Fc receptor (CD16/32, clone 2.4G2, BD Bioscience) in 1 ml PBS + 5% FCS for 10 min on ice to prevent antibody binding to Fc receptor. The isolated cells were stained on ice with various fluorescent monoclonal antibodies (mAbs) combinations in PBS + 5% FCS for 30 min. Anti-Ly6G-FITC (clone 1A8, BioLegend), anti-CD18-PE (clone M18/2, eBioscience), anti-CD11b-PECy7 (clone M1/70, BD Biosciences), anti-Ly6C-APC (clone AL-21, BD Biosciences), anti-CD62L-APCCy7 (clone MEL14, eBioscience), anti-MHCII-BV421 (clone M5/114.15.2, BD Biosciences), and anti-CD45-BV510 (clone 30-F11, BD Biosciences). A live/dead near-IR stain (ThermoFischer Scientific) was performed in PBS only. Unbound mAbs were discarded by washing the samples with PBS + 5% FCS and centrifugation (1,400 rpm, 7 min, 4°C). Analyses were performed using a FACS Canto II flow cytometer (BD Biosciences) and data were processed using FlowJo software (Tree Star Inc.). Cells were gated according to size and scatter to eliminate debris from analysis.

### Cytospins

Samples were cytocentrifuged using 100 µl of cell suspension per cuvette and spun at 500 rpm for 5 min. Cytocentrifuge slides were fixed and stained with Diff-Quick (Dade Behring, Deerfield, IL, USA). Slides were then assessed microscopically and differential cell counts were obtained by examining at least 500 cells.

### Real-Time PCR

Footpads were snap-frozen with liquid nitrogen and stored in cryotubes at −80°C. The samples were homogenized in 2 ml of Trizol reagent (ThermoFischer Scientific) with a Kontes^®^ Duall^®^ glass tissue grinder and total RNA was extracted with chloroform and precipitated with isopropanol-ethanol. DNA-free RNA was reverse transcribed to generate cDNA with oligo(dT) (25 µg/ml) in the presence of 500 nM dNTP, 200 U of Superscript II reverse transcriptase, 40 U of RNaseOut, 10 mM DTT, and First-strand Buffer (all reagents from Invitrogen). Real-time qPCR was performed on the CFX Connect™ (Bio-Rad). Relative quantities of mRNA for several genes were determined using SYBR Green (Bio-Rad) and by the comparative threshold cycle method, as described by the manufacturer. mRNA levels for each sample were normalized to ribosomal protein S11 gene (RPS11). Primers were used as described elsewhere (12); Rps11, forward, 5-CGTGACGAA GATGAAGATGC-3′ and reverse, 5-GCACATTGAATCGCACAGTC-3; Krt14, forward, 5′-ATCGAGGACCTGAAGAGCAA-3′ and reverse, 5′-TCGATCTGCAGGAGGACA TT-3′; Krt6a, forward, 5′-GAGGAGAGGGAGCAGATCAA-3′ and reverse, 5′-CACTTGGT GTCCAGGACCTT-3′; and Krt16, forward, 5′-TTGAGGACCTGAAGAGCAAGA-3′ and reverse, 5′-CCTGGCATTGTCAATCTGC-3′.

### Statistical Analysis

Statistical analysis was performed using Student’s *t*-test with GraphPad Prism software (GraphPad 6, San Diego, CA, USA). Values are expressed as mean ± SD values of *p* ≤ 0.05 are considered to be statistically significant.

## Results

### In the Absence of IL-12p40 and STAT6 Signaling, BALB/c Mice Display Reduced Footpad Swelling

**Figure 1:**
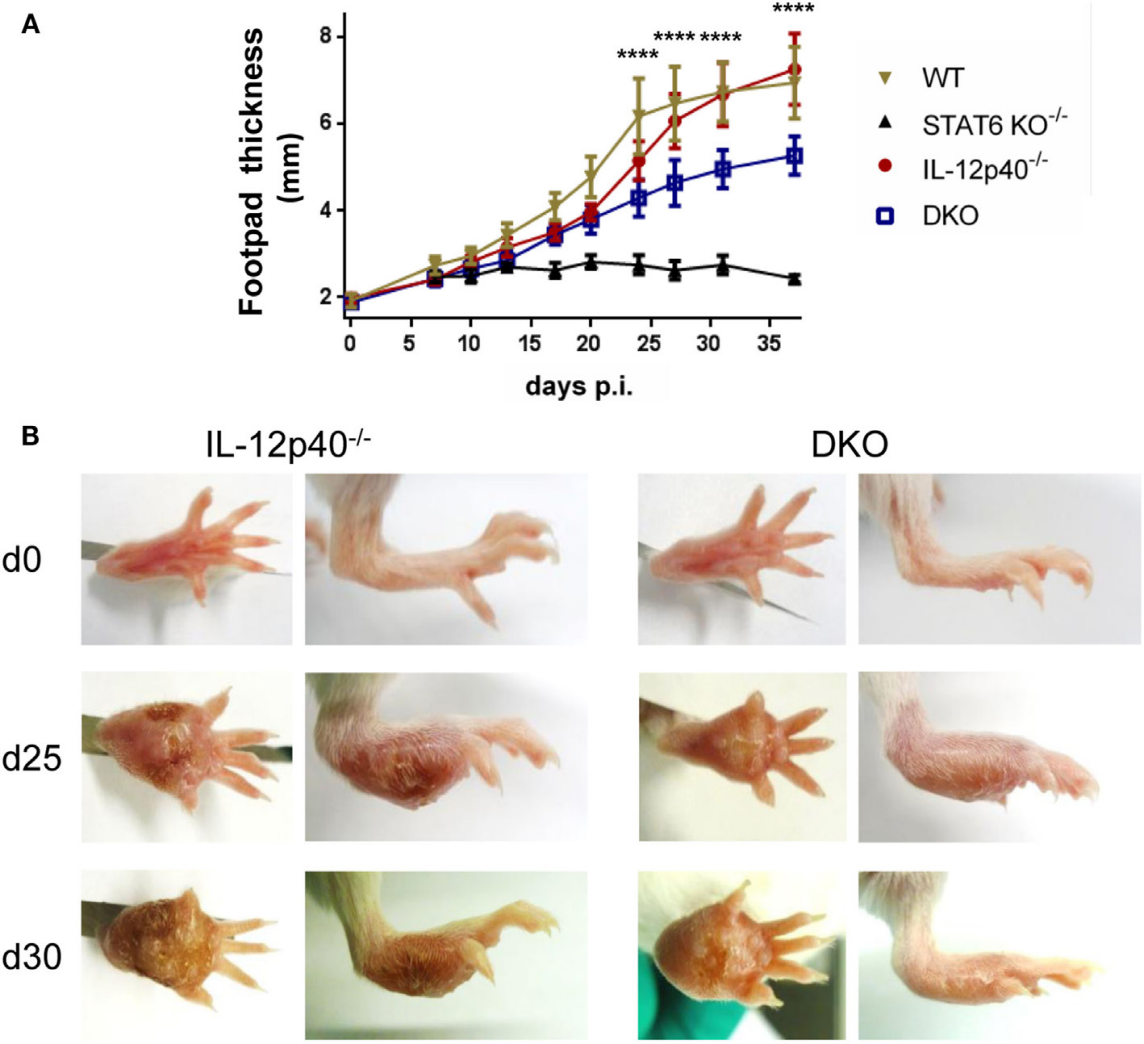
In the absence of IL-12p40 and STAT6 signaling, BALB/c mice display reduced footpad swelling. Footpad thickness measurements of naïve and *Leishmania major*-infected wild type (WT), IL-12p40^−/−^, STAT6^−/−^, and double knockout (DKO) BALB/c mice over the course of infection. **(A)** Footpad thickness (in millimeter) measured with a dial caliper at different days *p*.*i* and significant difference (*p*-value) displayed between IL-12p40^−/−^ and DKO mice. **(B)** Front and side view of the footpads of uninfected and infected mice at d25 and d30 post-infection for IL-12p40^−/−^ and DKO BALB/c mice. Data represent five independent experiments with at least six footpads per group. Error bars are shown as SDs. *p < 0.05, **p < 0.01, ***p < 0.001, ****p < 0.0001.

Upon *L. major* infection, wild-type (WT) BALB/c developed increasingly swelling footpads and this swelling was plateauing round d25 post-infection (*p.i*.) (Figure 1A). From d25 *p.i*. onward, footpads of *L. major*-infected WT mice became visibly necrotic (Figure S1 in Supplementary Material). Footpad swelling in infected IL-12p40^−/−^ mice initially lagged behind infected WT mice, but by d25 *p.i*. footpads of IL-12p40^−/−^ mice were similar in thickness and visual aspect as infected WT mice (Figures 1A,B) as previously shown (16). STAT6^−/−^ mice developed minor footpad swelling upon *L. major* infection, which nevertheless persisted leading to chronically slightly swollen footpads (Figure 1A) (20). However, ulceration was never observed in the footpads of infected STAT6^−/−^ mice over the course of infection (Figure S1 in Supplementary Material). We observed that footpads of infected IL-12p40^−/−^ STAT6^−/−^ DKO mice developed significantly smaller footpad swelling than WT and IL-12p40^−/−^ mice, but significantly bigger than infected STAT6^−/−^ mice (Figure 1A). Ulcerative footpad lesions started to appear in DKO mice around d30 *p.i*. and mostly confined to the inner region of the footpad (i.e., at the location of the parasite inoculation), whereas this type of necrotic lesion was already detected in IL-12p40^−/−^ mice at d25 *p.i*. (Figure 1B). Treatment of infected DKO mice with recombinant murine IL-12p70 cytokine during first week *p.i*. drastically reduces footpad swelling and impairs the appearance of this ulcerative lesion compared to untreated DKO mice (Figure S2 in Supplementary Material). Together, these results indicate a detrimental role of STAT6 in the development of footpad lesions and ulceration in susceptible BALB/c mice following *L. major* infection.

### Wound Healing of the *L. major*-Infected Footpad Is Negatively Regulated by STAT6 Signaling

**Figure 2:**
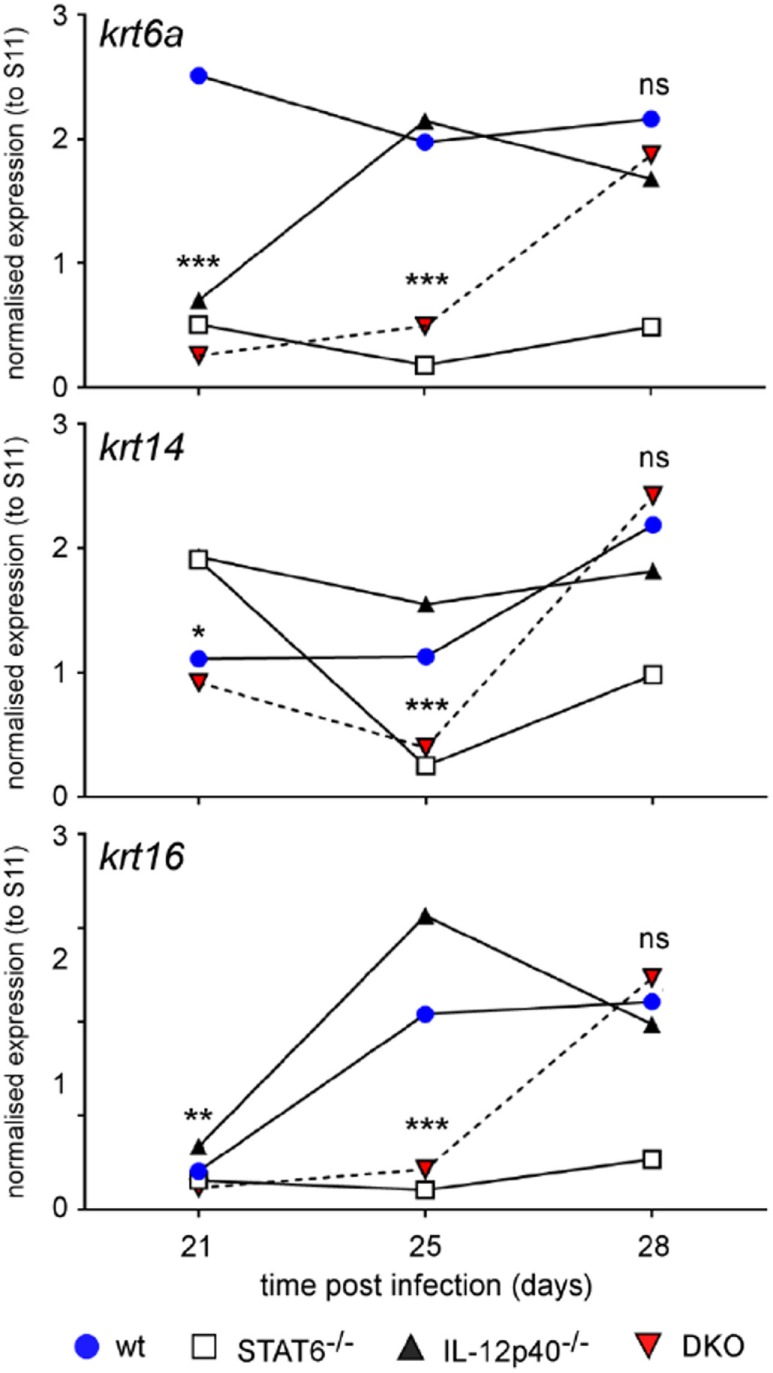
IL-12p40^−/−^ STAT6^−/−^ double knockout (DKO) mice show delayed expression of wound healing-associated keratinocyte markers. Wild type, IL-12p40^−/−^, STAT6^−/−^ and DKO BALB/c mice were infected with 2 × 10^6^ metacyclic promastigotes. RNA from naïve and infected mice was extracted from homogenized footpads at d21, d25, and d30 post-infection. Expression of the keratin 6a (krt6a), krt14, and krt16 genes were assessed and normalized to the expression of housekeeping gene *rps11*. Data are representative of two different experiments with at least four footpads per group.

One of the typical properties of *L. major*-induced wound is its capacity to self-heal (23). This phenomenon mainly depends on the keratinocyte differentiation and proliferation capacities (24). As we observed different footpad lesion patterns in the various deficient and wild-type mice, we decided to follow the impact of STAT6 and/or IL-12p40 signaling disruption on the expression of several keratinocyte-associated gene, such as Krt6a, Krt14, and Krt16, by real-time PCR in the footpads of *L. major*-infected mice at different time-points. Expression of Krt6a and Krt16 are usually upregulated in chronic wounds and can inhibit the potential of keratinocytes to properly heal wounds and damage (25, 26). As shown in Figure 2, on d21 *p.i*., all four infected groups expressed rather similar levels (within twofold) of Krt14 and Krt16. However, we observed that Krt6a expression was already higher in infected WT BALB/c compared to the other groups, which fits with lesion size of the footpad at this similar time point and the fact that IL-12p40^−/−^ BALB/c lagged behind infected WT BALB/c mice. On d25 *p.i*., infected WT and IL-12p40^−/−^ BALB/c displayed higher expression of Krt6a, Krt14, and Krt16 compared to infected STAT6^−/−^ and DKO BALB/c, suggesting that STAT6 is playing a detrimental role in wound healing. This result matched again with the lesion size observed in WT and IL-12p40^−/−^ BALB/c in Figure 1, where thickness and visual aspect of the footpads are similar in these two groups. On d28 *p.i*., we observed that infected DKO mice possessed a similar expression profile of keratins compared to infected WT and IL-12p40^−/−^ BALB/c, confirming the appearance of necrotic lesions in the DKO mice as shown in Figure 1. By contrast, infected STAT6^−/−^ mice exhibited low expression of these keratins at the three different time points tested, which fits with the absence of necrotic lesions in these mice. Together, these results suggest a detrimental role of STAT6 signaling in wound healing by upregulating the expression of keratins that inhibits wound and damage healing, thereby promoting footpad necrosis following *L. major* infection.

### Parasite Control and Footpad Pathology Are Independently Regulated in DKO Mice

**Figure 3:**
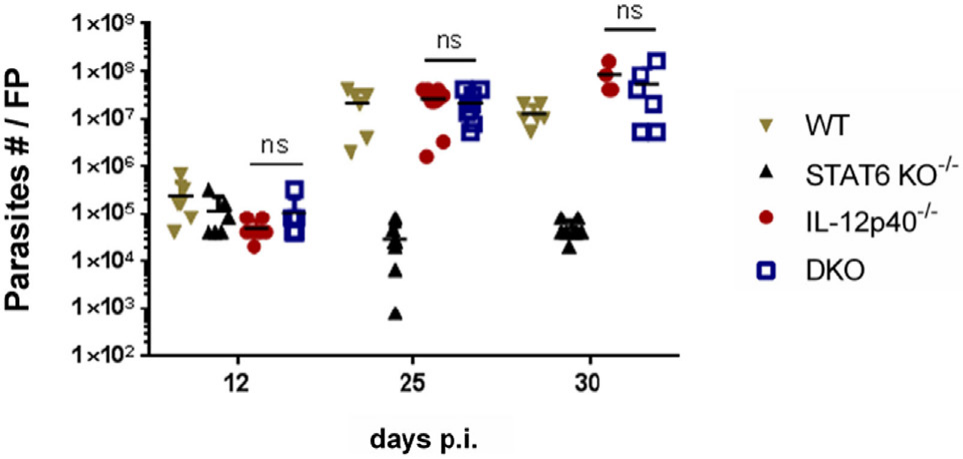
Impact of IL-12p40 and STAT6 on parasite control and footpad pathology are independently regulated. Parasitemia levels in the footpads of *Leishmania major*-infected wild type (WT), IL-12p40^−/−^, STAT6^−/−^, and double knockout (DKO) BALB/c mice at d12, d25, and d30 post-infection were obtained by the limiting dilution technique. Data represent three independent experiments with at least four footpads per group. Error bars are shown as SD. ns, not significant, *p < 0.05, **p < 0.01, ***p < 0.001.

Since footpads of DKO mice developed intermediate footpad swelling and delayed ulceration, the amount of parasites present in the footpads at three distinct time points was assessed by limiting dilutions. These time points were defined by a combination of footpad swelling and the presence of ulceration. The d12 *p.i* time point coincided with equal footpad swelling and the absence of footpad necrosis for all groups investigated (Figure 1; Figure S1 in Supplementary Material). The d25 *p.i*. time-point was characterized by significant differences in footpad swelling between the DKO mice and the single knockout mice, but with the absence of apparent footpad ulceration in the DKO mice as opposed to necrotic footpads in infected WT and IL-12p40^−/−^ mice (Figure 1; Figure S1 in Supplementary Material). The d30 *p.i*. time-point was defined by the presence of ulcerative footpad lesions in infected DKO mice (Figure 1; Figure S1 in Supplementary Material). At d12 *p.i*., no significant differences were observed in the footpad parasite burden between all groups (Figure 3). At d25 *p.i*., footpad parasite burdens of WT, IL-12p40^−/−^, and DKO mice were not significantly different from each other but significantly higher than in STAT6^−/−^ mice (Figure 3). At d30 *p.i*., footpads of infected IL-12p40^−/−^ and DKO mice contained a higher amount of parasites than infected WT mice (Figure 3). As on d25 *p.i*., footpads of infected STAT6^−/−^ mice had the lowest parasite levels on d30 *p.i*. (Figure 3). In summary, these results suggest that the footpad lesion size does not correlate with the associated parasite load.

### Neutrophils in IL-12p40^−/−^ STAT6^−/−^ Mice Express Lower Levels of Ly6C, CD11b, and Ly6G Markers

**Figure 4:**
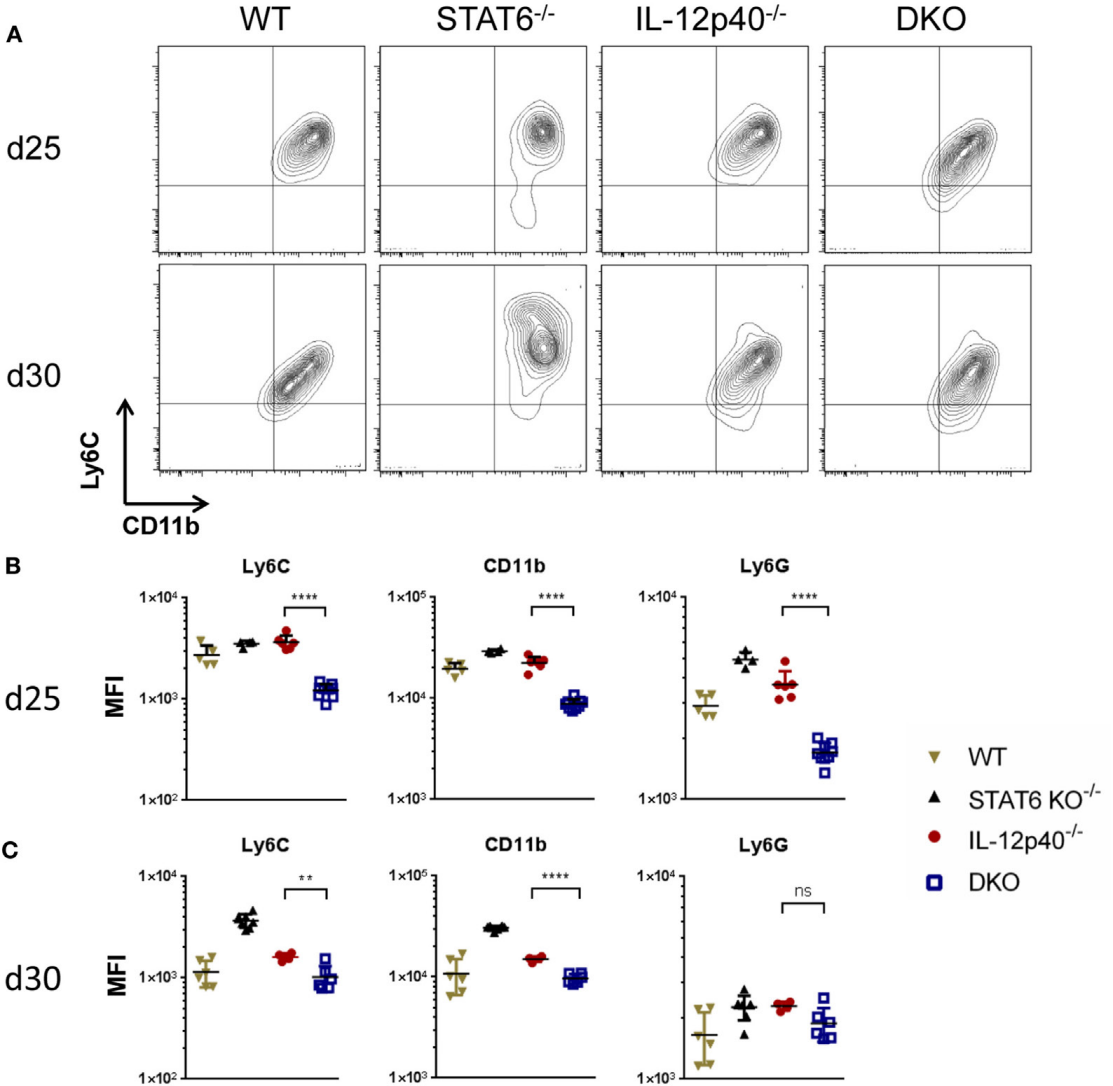
Neutrophils in IL-12p40^−/−^ STAT6^−/−^ double knockout (DKO) mice express lower levels of Ly6C, CD11b, and Ly6G. Wild type, IL-12p40^−/−^, STAT6^−/−^, and DKO BALB/c mice were infected with 2 × 10^6^ metacyclic promastigotes. Footpads of naïve and infected mice at d25 post-infection (*p.i*.) **(A,C)** and d30 *p.i*. **(B,C)** were homogenized and analyzed by flow cytometry as explained in Figure S2 in Supplementary Material. Ly6G^+^ neutrophils were plotted on Ly6C versus CD11b axes **(A,B)** and their MFI for Ly6C, CD11b, and Ly6G calculated **(B,C)**. Data represent three independent experiments with at least four footpads per group. Error bars are shown as SD. **p < 0.01, ****p < 0.0001.

**Figure 5:**
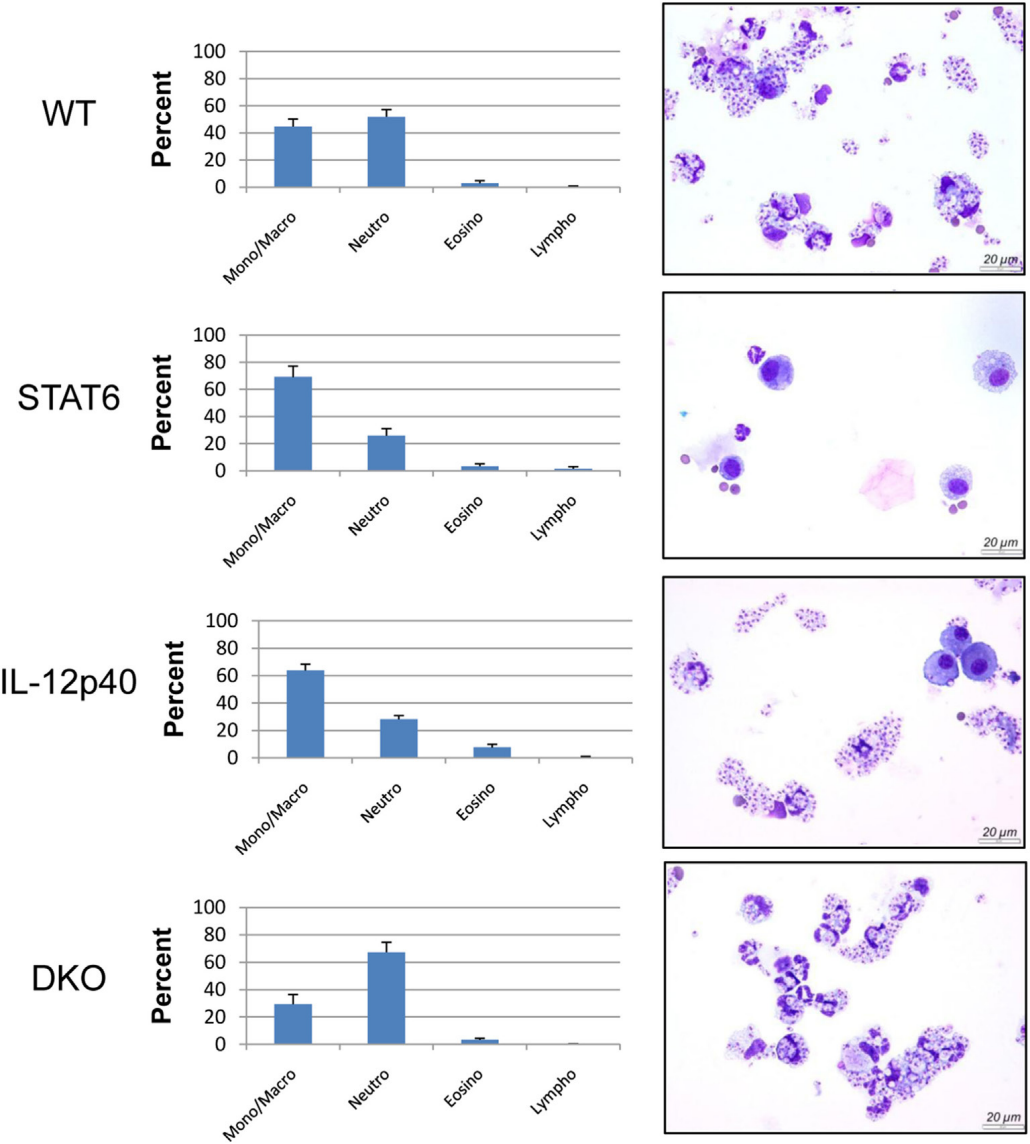
Majority of the cells present in double knockout (DKO) mice were neutrophils despite their altered phenotype. Wild type, IL-12p40^−/−^, STAT6^−/−^, and DKO BALB/c mice were infected with 2 × 10^6^ metacyclic promastigotes. Footpads of infected mice at 25 post-infection were homogenized. Cytospins and Diff-Quick staining were performed and content was enumerated. Representative pictures of cytospins from the different groups were taken.

Considering the importance of neutrophils during *L. major* infection in generating pathology in BALB/c mice (14), a flow cytometric analysis was performed on the footpads of naïve and infected mice at d25 and d30 *p.i*. to characterize these cells. Neutrophils were gated based on size, granularity, viability, and the expression of CD45^+^ and Ly6G^hi^ (Figures S3A–E in Supplementary Material). Further characterization of these neutrophils indicated them as being Ly6C^int^ CD11b^hi^ MHCII^−^ SiglecF^−^ (Figure S2F in Supplementary Material). Other CD11b^+^ cells such as monocytes, macrophages, and eosinophils were also present at the site of infection. From the total CD45^+^ living cell population within the footpads, neutrophils constituted ±60% in WT mice, ±32% in STAT6^−/−^ mice, ±50% in IL-12p40^−/−^ mice, and ±70% in DKO mice. The expression of Ly6C, Ly6G, and CD11b on the surface of neutrophils have been reported to correlate with cell maturation and/or differentiation (27–29). The expression of the surface markers Ly6C, CD11b, and Ly6G on the neutrophils within the footpads of DKO mice was significantly lower than in IL-12p40^−/−^ mice at d25 *p.i*. (Figures 4A,B). At d30 *p.i*., the expression levels of Ly6C and CD11b on neutrophils in infected footpads are lower in all groups, except for STAT6^−/−^ mice, as compared to d25 *p.i*. (Figures 4A–C). Hence, neutrophils within footpads of DKO mice expressed lower levels of Ly6C and CD11b, but not Ly6G, compared to IL-12p40 mice at d30 *p.i*. (Figures 4A,C). However, despite their altered phenotype, Diff-Quick staining of cytospins from infected footpad at d25 *p.i*. confirmed that the majority of the cells present in DKO mice were neutrophils (Figure 5). Overall, these results show that the absence of STAT6 signaling in IL-12p40^−/−^ mice induces the recruitment of neutrophils that display an altered phenotype.

### Neutrophils From DKO Mice Exhibit Less Apoptosis

**Figure 6:**
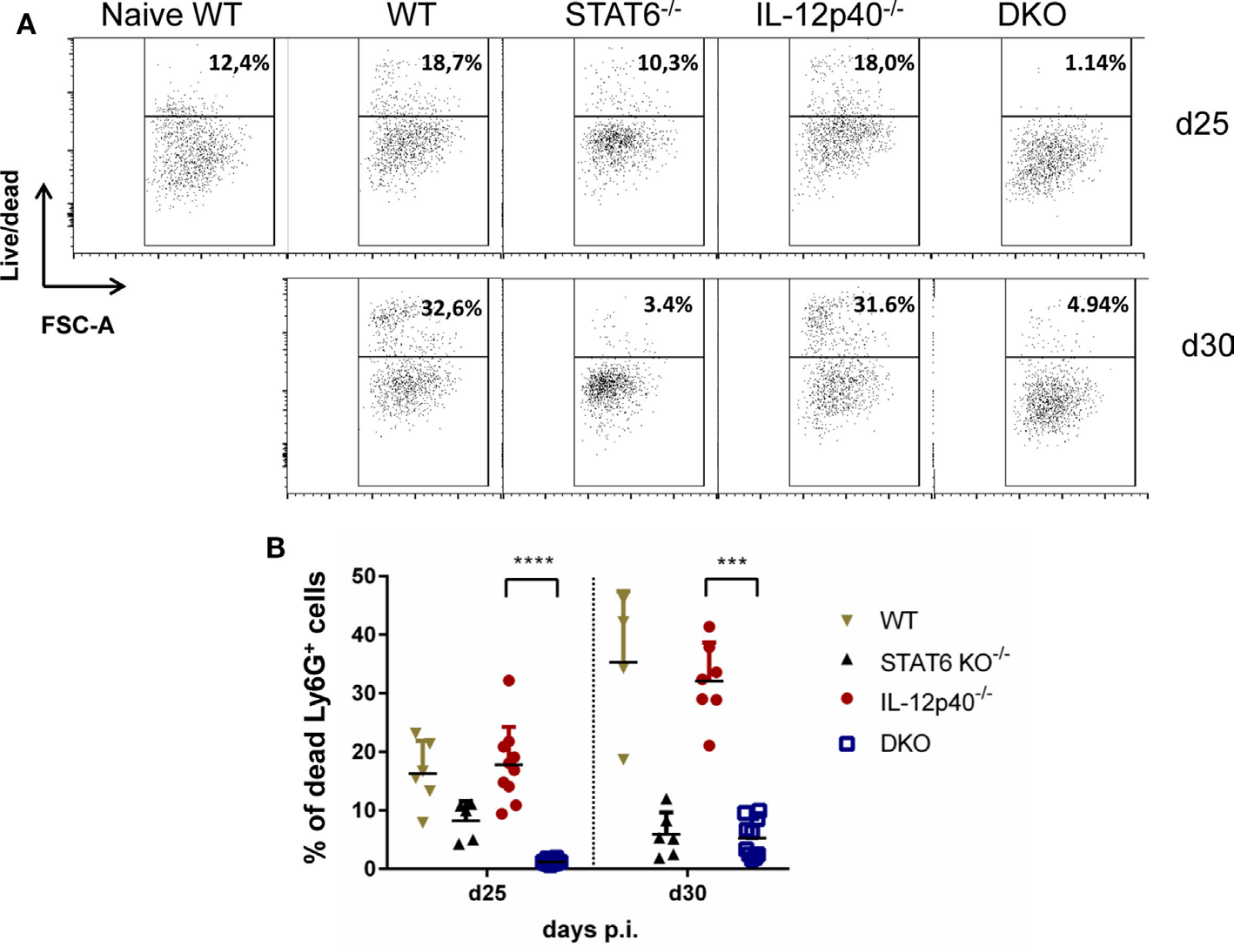
Neutrophils in IL-12p40^−/−^ STAT6^−/−^ double knockout (DKO) mice are less apoptotic. Wild type, IL-12p40^−/−^, STAT6^−/−^, and DKO BALB/c mice were infected with 2 × 10^6^ metacyclic promastigotes. Footpads of naïve and infected mice at d25 and d30 post-infection were homogenized and analyzed by flow cytometry as explained in Figure S2 in Supplementary Material after a live/dead staining. Ly6G^+^ neutrophils were plotted on **(A)** live/dead versus FSC-A axes and their **(B)** percentages of living Ly6G^+^ neutrophil population expressed. Data represent two independent experiments with at least four footpads per group. 1,000 cells per FACS plot are displayed for each group. Error bars are shown as SDs. ***p < 0.001, ****p < 0.0001.

As we noticed significant differences in footpad swelling and ulceration that correlate with an atypical neutrophil phenotype in infected DKO compared IL-12p40^−/−^ mice, we decided to look at the level of neutrophil apoptosis in these mouse strains. Interestingly, we observed that the proportion of dead neutrophils present in DKO mice at d25 and d30 *p.i*. was much lower than the one present in WT or IL-12p40^−/−^ mice (Figures 6A,B). Also, the percentages of apoptotic neutrophils inside the footpad of WT mice went from ±10% in naïve mice to ±15% at d25 *p.i*. to ±35% at d30 *p.i*., which exactly phenocopy the levels of neutrophil apoptosis observed in infected IL-12p40^−/−^ mice at both time-points (Figures 6A,B). Neutrophils within footpads of STAT6^−/−^ mice had intermediate levels of apoptosis (±10%) at d25 *p.i*. and this latter further decreased (±3%) at d30 *p.i*. to the levels observed in DKO mice (±5%) (Figures 6A,B). Similarly, the proportion of Annexin V^+^ alive neutrophils present in the footpad of DKO^−/−^ mice (±26%) is much lower compared to their counterpart observed in WT (±50%), STAT6^−/−^ (±50%), and IL-12p40^−/−^ (±70%) mice (Figure S4 in Supplementary Material). Together, these results suggested that the viability of the neutrophils present in the footpad of DKO mice was much higher compared to their profile in IL-12p40^−/−^ and wild-type mice, which correlates with less footpad swelling and ulceration.

## Discussion

Neutrophils have been shown to be important immune modulators and to induce pathology during *L. major* infections in susceptible mice (14). In the present study, we have investigated the combined effect of the absence of the Th1-associated IL-12p40 and of the Th2-associated STAT6 proteins on the immunopathology and neutrophil phenotype generated during *L. major* IR75 infection in the susceptible BALB/c mice model. BALB/c mice are intrinsically skewed toward a STAT6-mediated Th2 response (1). Upon infection with *L. major* IR75, STAT6^−/−^ BALB/c mice developed small lesion, as could be expected from previous reports on murine CL (20, 21). This result confirms the observation that BALB/c mice are able to mount a protective IFN-γ response upon *L. major* infection when given recombinant IL-12 (30, 31), but are unable to do so due to the inhibitory effect of STAT6. The absence of IL-12p40 did not have major impact on footpad lesions during *L. major* IR75 infection in BALB/c mice, as IL-12p40^−/−^ BALB/c mice developed similar lesions as infected WT BALB/c mice. Footpad swelling in DKO mice was significantly lower compared to susceptible WT and IL-12p40^−/−^ mice, but significantly higher compared to resistant STAT6^−/−^ mice. However, the treatment of infected DKO mice with recombinant IL-12 impaired footpad swelling and completely abolish the development of ulcerative lesions. Our results suggest that in the absence of both STAT6 and IL-12p40 signaling, BALB/c are still susceptible to *L. major* IR75 infection and develop susceptible pathological response, however, to a lesser extent than observed in infected WT and IL-12p40^−/−^ mice. Besides footpad swelling, the appearance of ulcerative lesions was also delayed in DKO mice compared to WT and IL-12p40^−/−^ mice and blocked in IL-12-treated DKO mice compared to untreated DKO mice.

Footpad pathology is generally correlated with the inability to control *L. major* parasites at the inoculation site, whereas healing is associated with immune activation of infected macrophages and the killing of parasites. In some cases of *L. major* infection, however, lesion development is dissociated from high parasite load (32). This uncommon observation can be related to the fact that the host potentially possesses two main ways to manage infection (33). One way, the most studied one, relies on the activation of immune effectors which will ultimately limit the pathogen burden. The other one mainly depends on the capacity of the host to limit immunopathology *via* its capacity to tolerate the infectious agent. Here, similar observations were done for both the single knockout mice, where STAT6^−/−^ mice had low parasite load at the site of inoculation and IL-12p40^−/−^ mice had comparable or higher footpad parasite loads than infected WT mice at both time points investigated. In the absence of both IL-12p40 and STAT6 signaling, parasite loads within the footpads of DKO mice were as high as in susceptible IL-12p40^−/−^ mice. These results confirm that STAT6 negatively regulates the control of parasitemia and that IL-12p40 signaling helps in controlling parasite replication at the later time point, but only to a limited extent. Moreover, these results suggest that the mechanisms which lead to STAT6 or IL-12p40-associated footpad pathology are independent from parasite control. Interestingly, DKO mice seem to better tolerate *Leishmania* parasites than IL-12p40^−/−^ mice as they develop slower immunopathology, which also suggests a negative role of STAT6 signaling in both resistance and tolerance. Although their footpad parasite levels were the lowest of all groups, STAT6^−/−^ mice were nevertheless not able to fully clear out *L. major* IR75 parasites from their footpads.

Neutrophils are crucial actors during *L. major* infections. They are not only the first cells to arrive at the site of infection to interact with the parasites (8) but also play important immunomodulatory functions leading to the activation of an immune response being disease promoting or controlling (34, 35). Moreover, *L. major* infection induces distinct neutrophil phenotypes depending on the genetic background of the infected murine host (35). Here, we report that in the absence of both STAT6 and IL-12p40 signaling, neutrophils within the footpads of DKO mice, display altered phenotype based on their lower expression of Ly6C, CD11b, and Ly6G. A low expression of Ly6G on neutrophils has been associated with immature neutrophils (28, 36). The expression of Ly6C and CD11b on monocytes can be modulated upon maturation during infection or inflammation (37, 38). However, cytospin analysis of these neutrophils within the footpads of infected DKO mice revealed segmented nucleus formation, suggesting that these neutrophils were mature (Figure 5). Investigating the presence of neutrophil extracellular traps or the cytokines produced by these neutrophils present in the footpads of DKO mice might provide information as to what their function is. The active phase of CL, i.e., when ulcerative lesions are present on the skin, is characterized by apoptosis and necrosis of keratinocytes (39, 40). Phagocytic clearance of apoptotic neutrophils is a common mechanism to control inflammation (41). During *L. major* infection, infected apoptotic neutrophils surrounding necrotic keratinocytes have been shown to act as a vehicle for the subsequent parasite uptake by macrophages analogous to the “Trojan Horse” (22, 34, 42, 43). Apoptotic cells that cannot be removed by phagocytic cells progress to a secondary necrotic stage (41). Necrosis, in which cells release their content in the environment and induce an inflammatory response, has been less documented for neutrophils during CL (42). Upon *L. major* IR75 infection, neutrophils within footpads of WT BALB/c mice showed an increasing level of necrosis reaching up to ±35% at d30 *p.i*. Susceptible IL-12p40^−/−^ mice had similar levels of neutrophil necrosis as WT mice. Interestingly, STAT6^−/−^ and DKO mice had low levels of neutrophil necrosis upon *L. major* infection. This might explain why the physiological aspect of footpads from DKO mice was less ulcerative. Additionally, these results suggest a negative regulation of STAT6 signaling on neutrophil necrosis upon *L. major* infection, since its absence coincided with low levels of neutrophil necrosis as observed in STAT6^−/−^ mice and DKO mice. Together, the results also showed a correlation between the presence of less apoptotic neutrophils with an altered phenotype in the footpad of DKO mice and the increased tolerance to *L. major* infection in these mice compared to IL-12p40^−/−^ mice, which reinforce a possible role of STAT6 in promoting immunopathology. To resolve *L. major*-induced footpad lesions, resistant mice activate a skin repair mechanism similar to wound healing, which requires the proliferation and activation of keratinocytes (24, 44, 45). Keratin filaments are essential components during wound repair as they supply the necessary cell and tissue strength during epithelial movements. Krt6a is a wound-induced protein which has been shown to negatively regulate keratinocyte migration (26, 46). In the context of *L. major* infections, increased Krt6a gene expression has been associated with increased lesion pathology (47). IL-12p40^−/−^ mice had similar levels of Krt6a expression as WT mice upon *L. major* infection. Interestingly, footpads of DKO mice had similarly low levels of Krt6a as observed in STAT6^−/−^ mice at d25 *p.i*. These results suggest that keratinocyte migration in STAT6^−/−^ mice and DKO mice are less inhibited, which in turn might explain the observed footpad phenotype in infected STAT6^−/−^ mice and DKO mice, being less ulcerative than in IL-12p40^−/−^ mice at d25 *p.i*. and could also explain their higher tolerance. However, at d28 *p.i*., krt6a gene expression in DKO mice was similar to its level in IL-12p40^−/−^ mice suggesting that this phenomenon is transient. Indeed, DKO mice start developing ulcerative lesions as of d30 *p.i*. localized in the center of the footpads at the site of parasite injection. These results also define a previously undescribed negative role for STAT6 on wound healing during *L. major* infections.

In conclusion, this report shows that in the absence of both STAT6 and IL-12p40 signaling, *L. major*-infected BALB/c mice remain susceptible to infection but demonstrate higher tolerance to pathogenic threat as they display smaller footpad lesions compared to IL-12p40^−/−^ mice. Footpads of DKO mice present neutrophils with lower Ly6C, CD11b, and Ly6G expression, which have low levels of necrosis. Finally, footpads of DKO mice do not inhibit keratinocyte migration during *L. major* IR75 infection. These results refine the roles of IL-12p40 and STAT6 on footpad pathology, parasite control, neutrophil necrosis, and keratinocyte migration during *L. major* IR75 infection in the BALB/c model and add more insights into the resistance and tolerance mechanisms required to survive a pathogenic infection.

## Ethics Statement

All the experiments were performed according to directive 2010/63/EU of the European Parliament for the protection of animals used for scientific purposes and approved by the Ethical Committee for Animal Experiments of the Vrije Universiteit Brussel (clearance number 14-220-18).

## Author Contributions

ERM and CDT conceived and designed the experiments; FK, ELM, KJ, YE and OD performed the experiments; DM contributed to tool development. FK, ELM, KJ and CDT analysed the data. ERM, SM and CDT supervised the work. FK, ERM, SM and CDT wrote the article.

## Conflict of Interest Statement

The authors declare that the research was conducted in the absence of any commercial or financial relationships that could be construed as a potential conflict of interest. The reviewer MB and handling editor declared their shared affiliation.
