# Supplementary figures and images for: STAT6 Mediates Footpad Immunopathology in the Absence of IL-12p40 Following Infection of Susceptible BALB/c Mice With Leishmania major
Source: Front Immunol. 2018 Mar 14;9:503. doi: 10.3389/fimmu.2018.00503 (PMC5861353; doi:10.3389/fimmu.2018.00503)

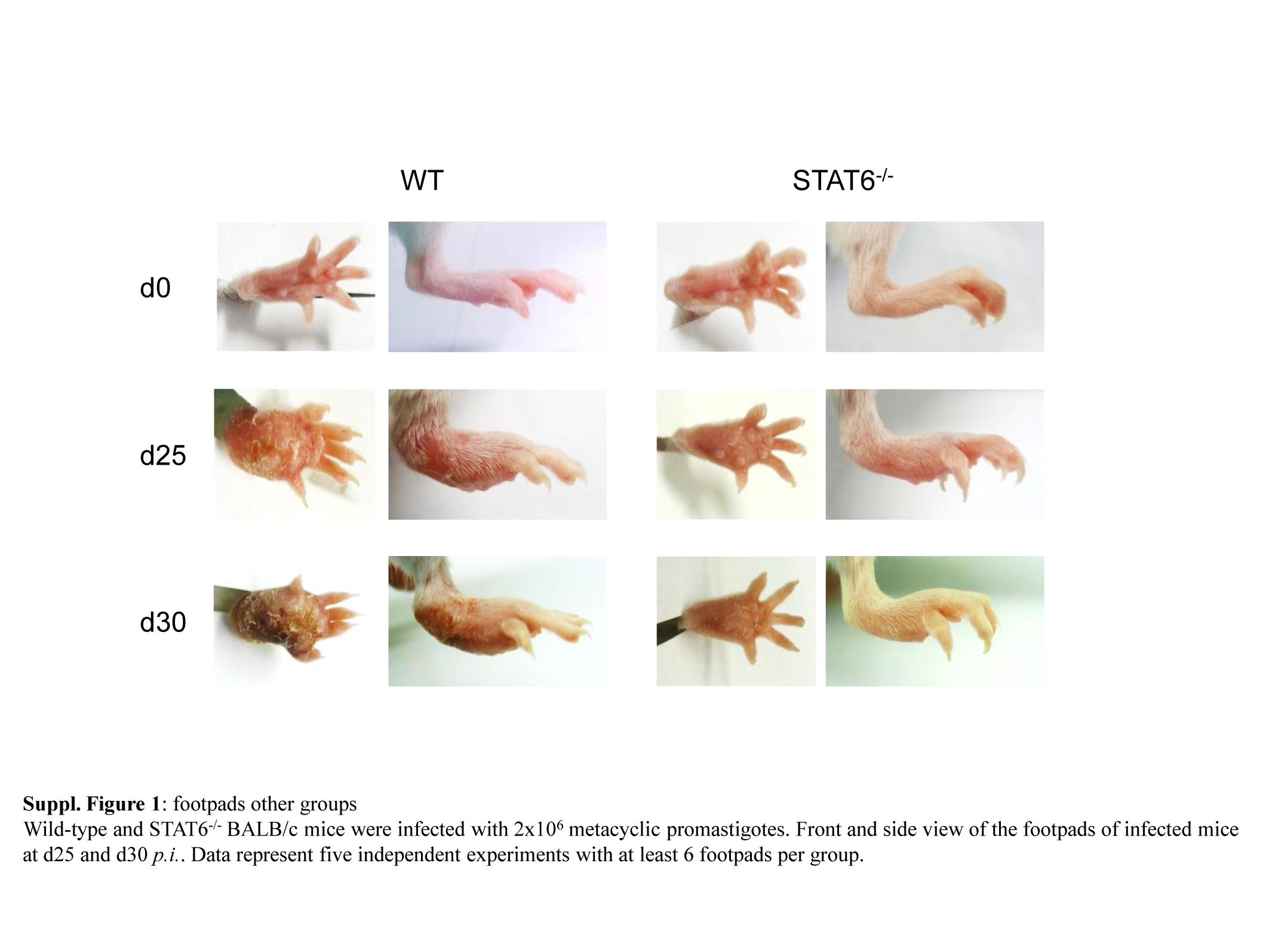

Supplement: Supplementary file 1 [file image_1.tif]

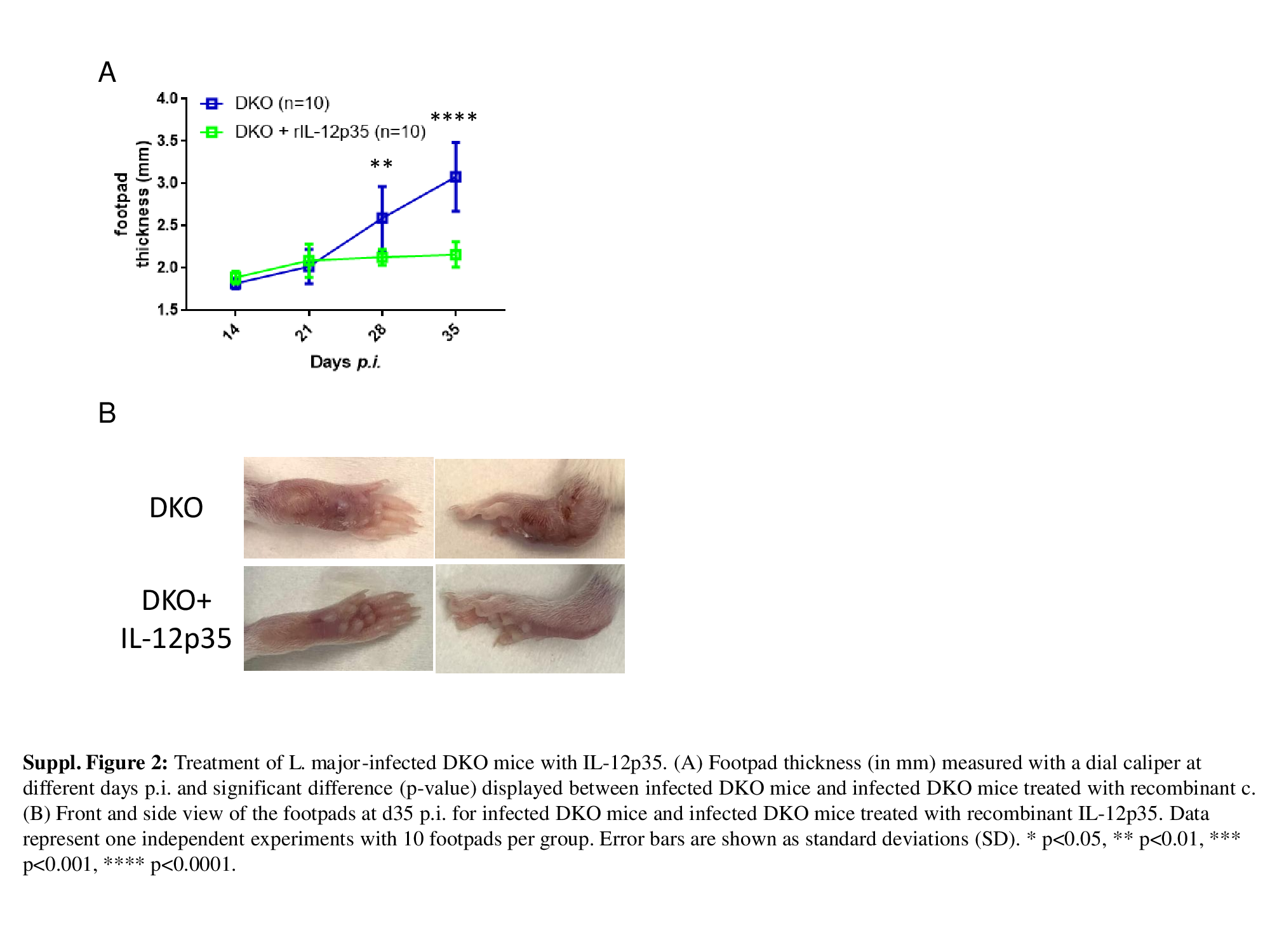

Supplement: Supplementary file 2 [file image_2.tiff]

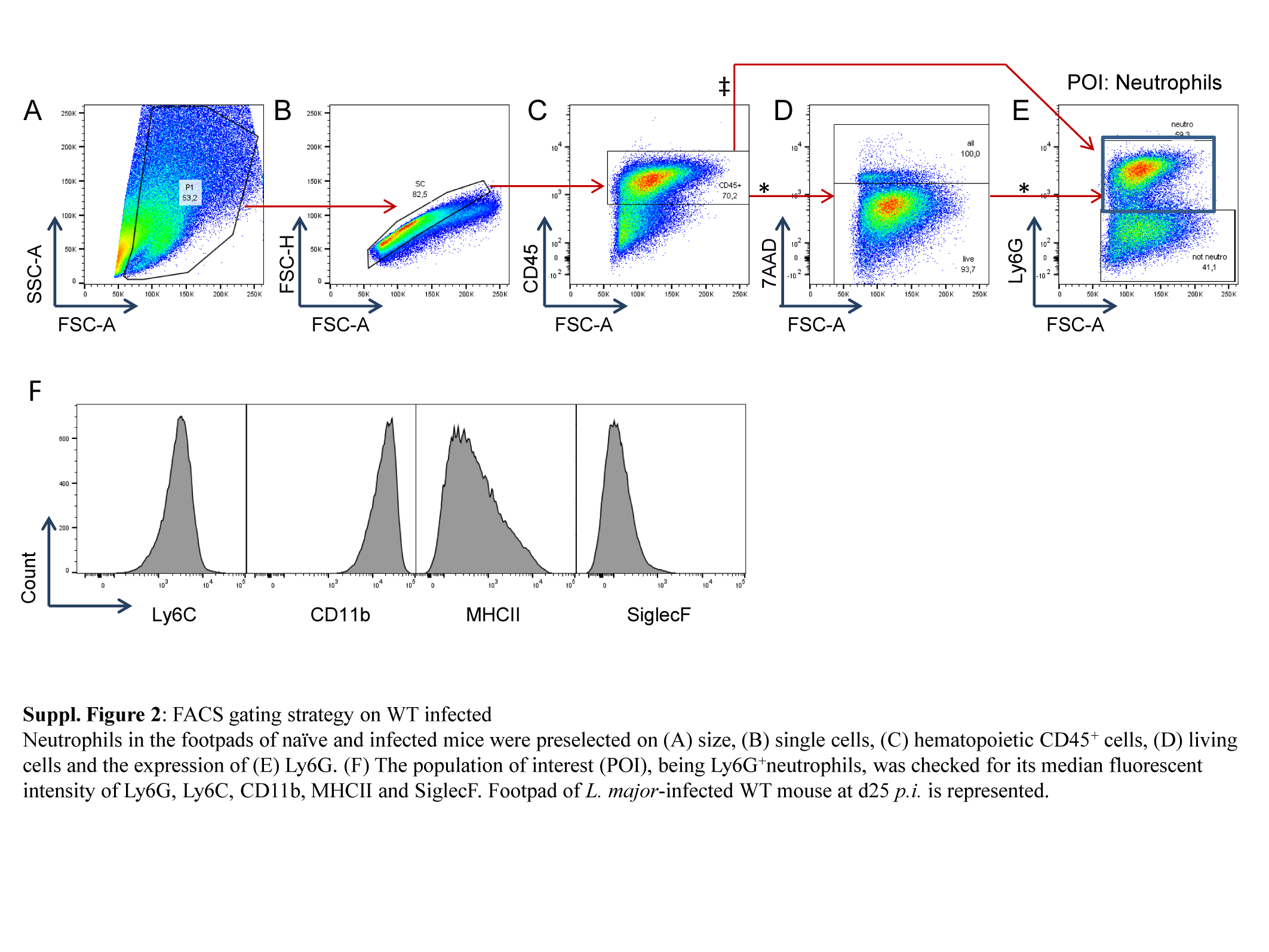

Supplement: Supplementary file 3 [file image_3.tif]

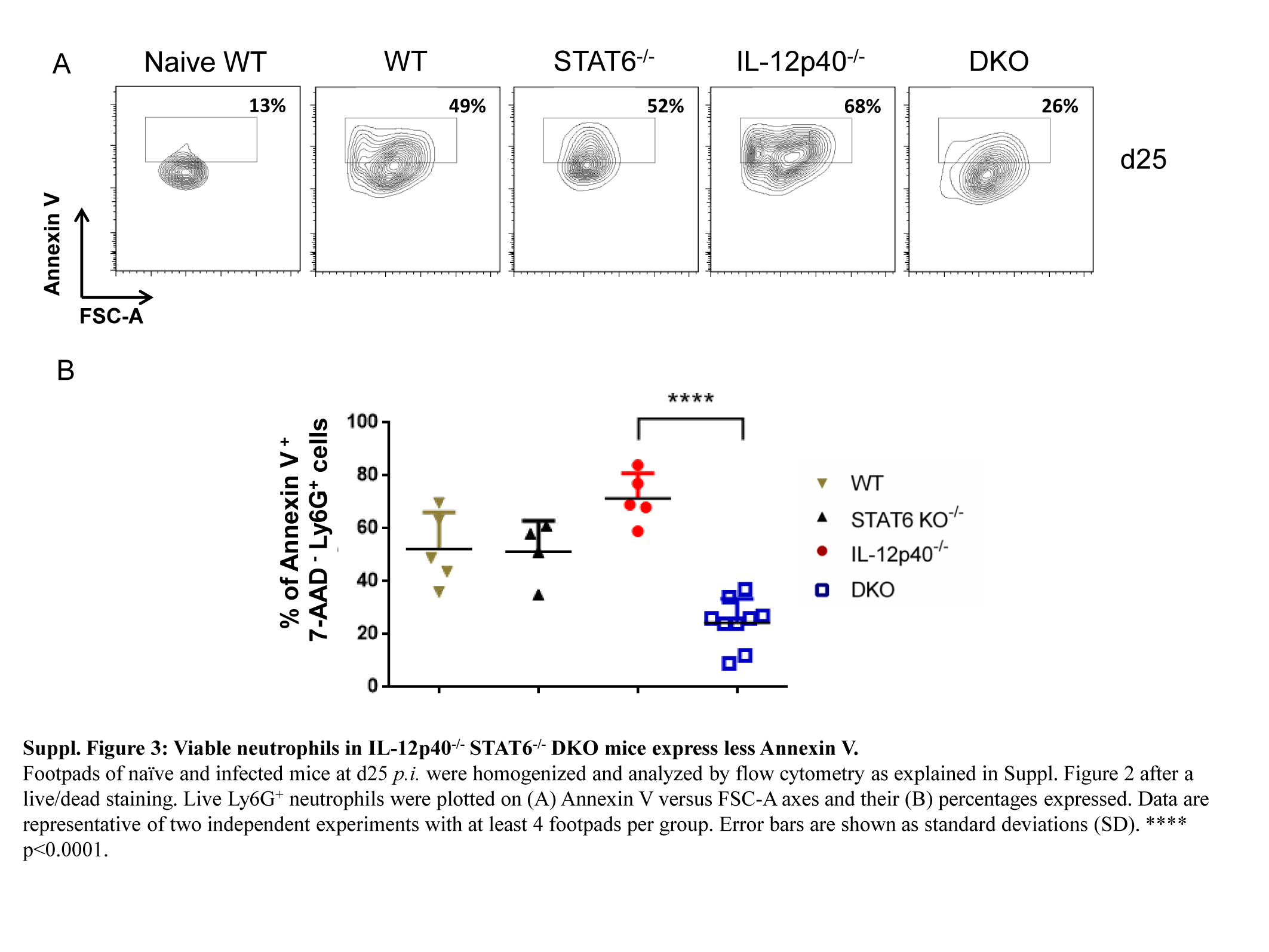

Supplement: Supplementary file 4 [file image_4.tif]
